# Vaccine Effectiveness against DS-1–Like Rotavirus Strains in Infants with Acute Gastroenteritis, Malawi, 2013–2015

**DOI:** 10.3201/eid2509.190258

**Published:** 2019-09

**Authors:** Khuzwayo C. Jere, Naor Bar-Zeev, Adams Chande, Aisleen Bennett, Louisa Pollock, Pedro F. Sanchez-Lopez, Osamu Nakagomi, Jacqueline E. Tate, Umesh D. Parashar, Robert S. Heyderman, Neil French, Miren Iturriza-Gomara, Nigel A. Cunliffe

**Affiliations:** University of Malawi, Blantyre, Malawi (K.C. Jere);; Malawi-Liverpool-Wellcome Trust Clinical Research Programme, Blantyre (K.C. Jere, N. Bar-Zeev, A. Chande, A. Bennett, L. Pollock, R.S. Heyderman, N. French, M. Iturriza-Gomara);; University of Liverpool, Liverpool, UK (K.C. Jere, N. Bar-Zeev, A. Bennett, L. Pollock, N. French, M. Iturriza-Gomara, N.A. Cunliffe);; Johns Hopkins Bloomberg School of Public Health, Baltimore, Maryland, USA (N. Bar-Zeev);; University of Murcia, Murcia, Spain (P.F. Sanchez-Lopez); Nagasaki University, Nagasaki, Japan (O. Nakagomi, J.E. Tate);; Centers for Disease Control and Prevention, Atlanta, Georgia, USA (U.D. Parashar);; University College London, London, UK (R.S. Heyderman)

**Keywords:** rotavirus, vaccine effectiveness, genome constellation, genotyping, whole-genome sequencing, viruses, vaccines, Malawi, infants

## Abstract

Atypical DS-1–like G1P[8] rotaviruses emerged in 2013 in Malawi after rotavirus vaccine introduction. Vaccine effectiveness among infants hospitalized with acute DS-1–like G1P[8] rotavirus gastroenteritis was 85.6% (95% CI 34.4%–96.8%). These findings suggest that vaccine provides protection against these strains despite their emergence coinciding with vaccine introduction.

Rotavirus remains a major cause of severe dehydration, diarrhea, and death among children <5 years of age in many low-income countries. After the introduction of Rotarix (Glaxo SmithKline, https://www.gsksource.com) rotavirus vaccine into Malawi’s immunization schedule in October 2012, enhanced surveillance combined with case–control studies have described the substantial population impact and effectiveness of Rotarix on hospitalized rotavirus disease and diarrheal deaths (*1*,*2*).

Both of the globally available rotavirus vaccines, Rotarix and RotaTeq (Merck & Co., https://www.merckvaccines.com), have been shown to protect against rotaviruses with a broad range of G and P types, as defined by the 2 viral outer-capsid proteins (*3*,*4*). A whole-genome classification system describes rotavirus strains more completely by assigning genotypes to each of its 11 double-stranded RNA segments (*5*). Most rotavirus strains contain either a Wa (G1-P[8]-I1-R1-C1-M1-A1-N1-T1-E1-H1) or DS-1 (G2-P[4]-I2-R2-C2-M2-A2-N2-T2-E2-H2) genotype constellation (*6*). Typically, G1P[8] strains, including the Rotarix strain (RIX4414), possess a Wa-like genetic backbone, whereas G2P[4] strains generally have a DS-1–like genotype constellation (*6*). A switch in predominant rotavirus genotype from G1P[8] to G2P[4] after Rotarix introduction has been described in various settings (*7*,*8*), and higher vaccine effectiveness (VE) against G1P[8] strains has been described compared with G2P[4] in some settings (*2*,*9*). It is not known whether these changes in strain distribution and strain-specific differences in VE are related to differences in cross-protection afforded by the outer capsid proteins (G and P type) or to the distinct genetic backbones possessed by DS-1–like strains and the Wa-like Rotarix strain.

Previously, we demonstrated that all G1P[8] strains detected before Rotarix introduction in Malawi had a Wa-like genetic backbone (*10*). Shortly after Rotarix introduction, atypical DS-1–like G1P[8] rotavirus strains were detected, which provided an opportunity to examine whether emergence of DS-1–like G1P[8] strains could be the result of reduced protection afforded by the Wa-like G1P[8] Rotarix vaccine.

## The Study

We used enzyme immunoassay (EIA) to detect rotaviruses in stool samples collected from children <5 years of age with acute gastroenteritis at Queen Elisabeth Central Hospital (QECH; Blantyre, Malawi) (*2*). We used reverse transcription PCR to assign G and P genotypes to rotavirus-positive samples (*10*,*11*). Samples with sufficient volume and containing G1 (n = 110), G2 (n = 64), or other (G4, G9, or G12, n = 42) rotavirus strains were selected at random during January 2013–December 2015.

We generated rotavirus whole-genome sequences (WGS) using the HiSeq 2000 platform (Illumina Inc., https://www.illumina.com) as described previously (*10*). We derived consensus sequences using Geneious (https://www.geneious.com) and genotyped them using RotaC (http://rotac.regatools.be). All complete nucleotide sequences generated in this study were deposited into GenBank (*12*) (accession nos. MG181227–941).

We calculated rotavirus VE using logistic regression to compare 2-dose versus 0-dose vaccination status among hospitalized strain-specific rotavirus diarrhea case-patients and concurrently hospitalized control patients with non–rotavirus-caused diarrhea, matched by age at admission. We defined concurrency of controls for each endpoint (Table) as any patient hospitalized for diarrhea who tested negative for rotavirus occurring in the same date range (between the first and last hospitalized strain-specific case) in which cases of strain-specific rotavirus were detected. We limited VE analysis to infants <12 months of age because previous analysis did not demonstrate statistically significant protection in the second year of life (VE 31.7%, 95% CI −140.6% to 80.6%) (*2*). We obtained ethics approval from the National Health Sciences Research Committee, Malawi (867), and the Research Ethics Committee of the University of Liverpool, Liverpool, UK (000490).

**Table:** Point estimates of vaccine effectiveness by rotavirus genotype constellation based on the complete genetic composition of rotavirus strains, Blantyre, Malawi*

Rotavirus strain type	Sequenced strains from test-positive case-patients†				Rotavirus test-negative controls				Adjusted logistic regression for year of presentation	
	No. tested positive	No. known vaccine status	No. (%) 2-dose vaccine		No. controls	No. known vaccine status	No. (%) 2-dose vaccine		Rotarix vaccine effectiveness, % (95% CI)	p value
DS-1–like G1P[8]	13	13	10 (76.9)		426	410	365 (89)		85.6 (34.4–96.8)	0.01
DS-1–like G2	30	28	24 (85.7)		481	465	411 (88.4)		48.5 (−154.3 to 89.6)	0.42
Wa-like G1	38	38	34 (89.5)		440	424	376 (88.7)		76.7 (−153.8 to 97.9)	0.23

*Rotavirus strains detected at Queen Elisabeth Central Hospital during January 2013–December 2015. Case-patients were fully vaccinated infants <12 mo of age. †Complete whole-genome sequences were generated.

Of 216 rotavirus strains sequenced, 114 (53%) had a Wa-like and 88 (44%) a DS-1–like genotype constellation. Among Wa-like strains, 72% were G1, <1% were G2, and 25% were G12. Of the DS-1–like strains, 31% were G1 and 69% were G2. Of the 110 G1 strains analyzed by WGS, 75% were Wa-like and 25% were DS-1–like. We detected atypical G1 rotaviruses with DS-1–like genotype constellation in Malawi in 2013; their circulation peaked in 2014 and subsequently decreased in 2015 (<1%, 1/72) (Figure).

**Figure:**
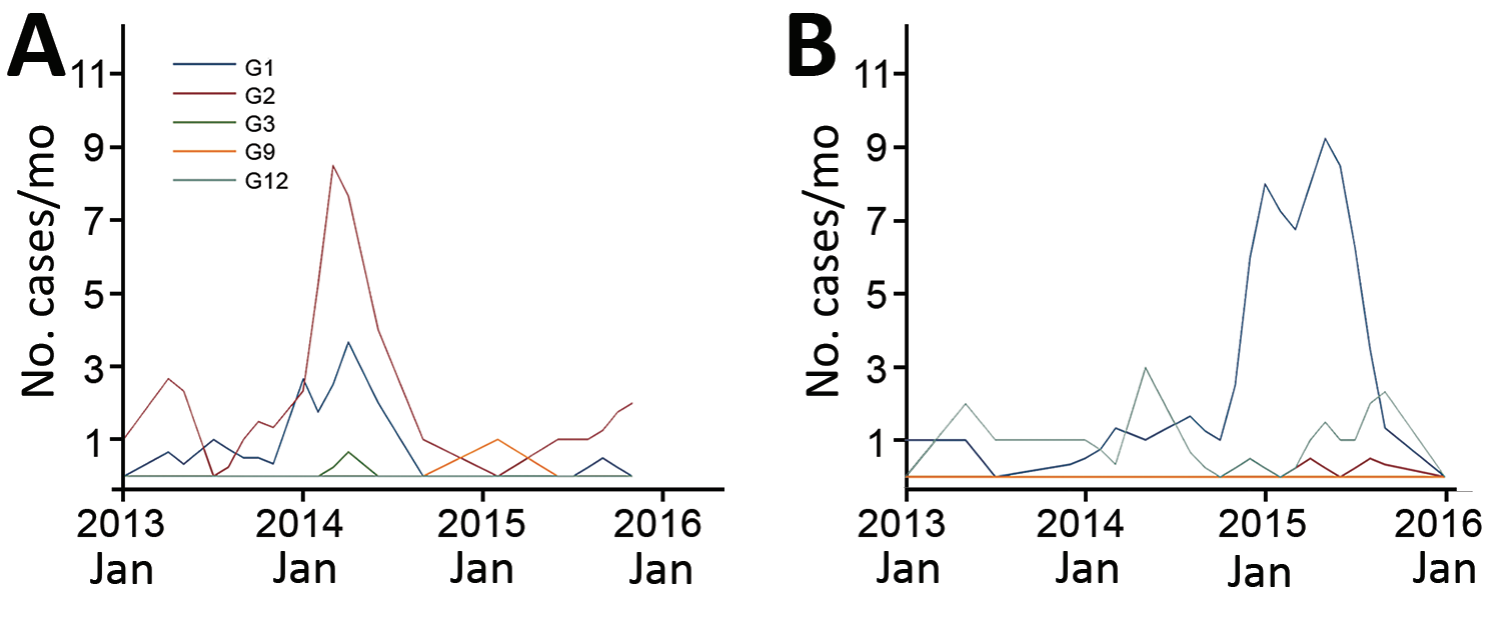
Monthly number of rotavirus cases at Queen Elisabeth Central Hospital, Blantyre, Malawi. Numbers are based on the presence of either DS-1–like (A) or Wa-like (B) constellation of rotavirus strains.

In logistic regression analysis adjusted for year of presentation, Rotarix effectiveness against DS-1–like G1P[8] rotavirus was 85.6% (95% CI 34.4%–96.8%; p = 0.01). Effectiveness estimates against Wa-like G1 (VE 76.7%, 95% CI −153.8% to 97.9%) and DS-1–like G2 (VE 48.5%, 95% CI −154.3% to 89.6%) rotaviruses included wide bounds and the null value (Table).

## Conclusions

Atypical DS-1–like G1 rotavirus strains emerged in Malawi shortly after Rotarix vaccine introduction (*10*). Although strain oscillation and emergence of novel types have been reported globally in the absence of vaccination, the mechanisms driving this phenomenon are not well understood. It is possible that the emergence of these DS-1–like G1P[8] strains was coincidental with vaccine introduction. The high VE strongly suggests that escape from vaccine-induced immunity is not the driver for emergence. The swift decline in prevalence of these strains is in contrast with more sustained changes in strain circulation described in other settings in the context of high VE (*13*). The decline could have been precipitated by the observed high VE or may represent a natural phenomenon related to viral fitness and associated periodic nature of the circulation of the DS-1–like strains, which has been observed historically and globally in the absence of vaccine. These findings support continued use of rotavirus vaccine in this population as an intervention to reduce severe diarrhea caused by rotavirus strains possessing either Wa-like or DS-1–like genetic backbones. The observed decline in rotavirus hospitalizations in children after vaccine introduction (*2*), together with reduction in infant diarrhea deaths in Malawi (*14*), are public health benefits that could be sustained through rotavirus vaccination in this region, which has one of the highest burdens of rotavirus disease.

The VE against DS-1–like G1P[8] strains in this study resembles our previous findings of VE of 82% (95% CI 42%–95%) against all G1P[8] strains 3 years after vaccine introduction (2013–2015) (*2*). In contrast, we were unable to demonstrate statistically significant VE against DS-1–like G2 rotaviruses despite a comparable number of such strains, consistent with our earlier study (VE 45.9%, 95% CI −47.0% to 80.1%; p = 0.228) (*2*). The apparently lower VE against rotavirus disease caused by DS-1–like strains associated with G2, but not with G1P[8], lends support to the proposed dominant role of the outer capsid proteins VP7 and VP4 as drivers of homotypic protection. Although increasing evidence suggests that Rotarix vaccine does not provide the same degree of protection against G2 strains as G1 strains, this difference in protection appears to have little effect on total VE among populations in which vaccination performs optimally and high VE is maintained. However, the difference in protection between the strains may exacerbate underperformance of rotavirus vaccines in low-resource settings such as Malawi, where overall VE is generally lower for reasons that remain poorly understood (*2*,*15*).

We could not demonstrate statistically significant effectiveness against Wa-like G1P[8] rotaviruses (p = 0.23). Wa-like G1P[8] cases became dominant and replaced DS-1–like G1P[8] once vaccine coverage had reached high and stable levels (Figure). At high population vaccine coverage, case–control analysis of VE became challenging and difficult to power sufficiently.

Our data demonstrate that Rotarix provides a high degree of protection against severe disease caused by homotypic G1P[8] rotaviruses in Malawi regardless of genomic backbone. VE for patients <1 year of age is comparable to that seen in middle-income countries. The lower VE against heterotypic G2P[4] strains previously described (*15*) suggests that more detailed immune response studies, clarification of the correlates of protection for rotavirus disease, and strain surveillance are needed to monitor the impact of sustained, high vaccine coverage on rotavirus strain distribution.

## References

[1] Tate JE, Burton AH, Boschi-Pinto C, Parashar UD; World Health Organization–Coordinated Global Rotavirus Surveillance Network. Global, regional, and national estimates of rotavirus mortality in children <5 years of age, 2000–2013. Clin Infect Dis. 2016;62(Suppl 2):S96–105. 10.1093/cid/civ1013 27059362 PMC11979873

[2] Bar-Zeev N, Jere KC, Bennett A, Pollock L, Tate JE, Nakagomi O, et al.; Vaccine Effectiveness and Disease Surveillance Programme, Malawi (VACSURV) Consortium. Population impact and effectiveness of monovalent rotavirus vaccination in urban Malawian children 3 years after vaccine introduction: ecological and case-control analyses. Clin Infect Dis. 2016;62(Suppl 2):S213–9. 10.1093/cid/civ1183 27059359 PMC4825885

[3] Vesikari T, Karvonen A, Prymula R, Schuster V, Tejedor JC, Cohen R, et al. Efficacy of human rotavirus vaccine against rotavirus gastroenteritis during the first 2 years of life in European infants: randomised, double-blind controlled study. Lancet. 2007;370:1757–63. 10.1016/S0140-6736(07)61744-9 18037080

[4] Zaman K, Dang DA, Victor JC, Shin S, Yunus M, Dallas MJ, et al. Efficacy of pentavalent rotavirus vaccine against severe rotavirus gastroenteritis in infants in developing countries in Asia: a randomised, double-blind, placebo-controlled trial. Lancet. 2010;376:615–23. 10.1016/S0140-6736(10)60755-6 20692031

[5] Matthijnssens J, Ciarlet M, Heiman E, Arijs I, Delbeke T, McDonald SM, et al. Full genome-based classification of rotaviruses reveals a common origin between human Wa-Like and porcine rotavirus strains and human DS-1-like and bovine rotavirus strains. J Virol. 2008;82:3204–19. 10.1128/JVI.02257-07 18216098 PMC2268446

[6] Matthijnssens J, Ciarlet M, McDonald SM, Attoui H, Bányai K, Brister JR, et al. Uniformity of rotavirus strain nomenclature proposed by the Rotavirus Classification Working Group (RCWG). Arch Virol. 2011;156:1397–413. 10.1007/s00705-011-1006-z 21597953 PMC3398998

[7] Zeller M, Rahman M, Heylen E, De Coster S, De Vos S, Arijs I, et al. Rotavirus incidence and genotype distribution before and after national rotavirus vaccine introduction in Belgium. Vaccine. 2010;28:7507–13. 10.1016/j.vaccine.2010.09.004 20851085

[8] Kirkwood CD, Boniface K, Barnes GL, Bishop RF. Distribution of rotavirus genotypes after introduction of rotavirus vaccines, Rotarix® and RotaTeq®, into the National Immunization Program of Australia. Pediatr Infect Dis J. 2011;30(Suppl):S48–53. 10.1097/INF.0b013e3181fefd90 21183840

[9] Braeckman T, Van Herck K, Meyer N, Pirçon JY, Soriano-Gabarró M, Heylen E, et al. RotaBel Study Group. Effectiveness of rotavirus vaccination in prevention of hospital admissions for rotavirus gastroenteritis among young children in Belgium: case-control study. BMJ. 2012;345(aug08 1):e4752. 10.1136/bmj.e4752 PMC3414434 22875947

[10] Jere KC, Chaguza C, Bar-Zeev N, Lowe J, Peno C, Kumwenda B, et al. Emergence of double- and triple-gene reassortant G1P[8] rotaviruses possessing a DS-1–like backbone after rotavirus vaccine introduction in Malawi. J Virol. 2018;92:e01246–17. 29142125 10.1128/JVI.01246-17 PMC5774894

[11] Cunliffe NA, Ngwira BM, Dove W, Thindwa BD, Turner AM, Broadhead RL, et al. Epidemiology of rotavirus infection in children in Blantyre, Malawi, 1997-2007. J Infect Dis. 2010;202(Suppl):S168–74. 10.1086/653577 20684698

[12] Benson DA, Karsch-Mizrachi I, Lipman DJ, Ostell J, Rapp BA, Wheeler DL. GenBank. Nucleic Acids Res. 2002;30:17–20. 10.1093/nar/30.1.17 11752243 PMC99127

[13] Hungerford D, Allen DJ, Nawaz S, Collins S, Ladhani S, Vivancos R, et al. Impact of rotavirus vaccination on rotavirus genotype distribution and diversity in England, September 2006 to August 2016. Euro Surveill. 2019;24. 10.2807/1560-7917.ES.2019.24.6.1700774 PMC6373066 30755297

[14] Bar-Zeev N, King C, Phiri T, Beard J, Mvula H, Crampin AC, et al. VacSurv Consortium. Impact of monovalent rotavirus vaccine on diarrhea-associated post-neonatal infant mortality in rural communities in Malawi: a population-based birth cohort study. Lancet Glob Health. 2018;6:e1036–44. 10.1016/S2214-109X(18)30314-0 30103981 PMC6088152

[15] Bar-Zeev N, Kapanda L, Tate JE, Jere KC, Iturriza-Gomara M, Nakagomi O, et al.; VacSurv Consortium. Effectiveness of a monovalent rotavirus vaccine in infants in Malawi after programmatic roll-out: an observational and case-control study. Lancet Infect Dis. 2015;15:422–8. 10.1016/S1473-3099(14)71060-6 25638521 PMC4374102

